# A Study of the Granulomatous Responses Induced by Different Strains of *Schistosoma mansoni*

**DOI:** 10.1155/2012/953524

**Published:** 2012-11-05

**Authors:** Nádia Regina Borim Zuim, Silmara Marques Allegretti, Arício Xavier Linhares, Luiz Augusto Magalhães, Eliana Maria Zanotti-Magalhães

**Affiliations:** Department of Animal Biology, Institute of Biology, State University of Campinas (UNICAMP), Cidade Universitária Zeferino Vaz, Rua Monteiro Lobato 255, 13083-862 Campinas, SP, Brazil

## Abstract

The increased pathogenesis of the *Schistosoma mansoni* BH strain compared with the SJ strain has been attributed to the number of granulomas formed in experimental infections, which increase the mortality in definitive hosts. The aim of the present study was to investigate the development of granulomas around the eggs of the *S. mansoni* BH and SJ strains and to determine whether this host reaction was strain specific. Four experimental groups were analyzed. Two groups contained mice inoculated in the caudal vein with eggs from the *S. mansoni* BH or SJ strains and the other two contained mice that were infected with cercariae of the BH strain prior to being inoculated with eggs. The number of granulomas per tissue area in the lungs and liver, as well as the size of the granulomas, was analyzed to characterize the response to schistosome infection. The largest granulomatous responses were observed around eggs of the BH strain. Granulomas covered a larger area in the lungs of mice that were previously infected with cercariae and subsequently inoculated with eggs of the BH strain. These results indicated that specific granulomatous responses occurred following an infection with the BH and SJ strains of *S. mansoni*.

## 1. Introduction

Schistosomiasis is considered to be the most notable parasitic disease after malaria due to its wide geographic distribution, the large number of people affected by the disease, its severity, and its association with poor sanitary conditions [1, 2].

The primary pathogenic mediators in schistosomiasis are the trematode eggs. A schistosomal granuloma, the characteristic lesion, forms around mature eggs that have been deposited in the tissues of the definitive host. The distribution of eggs in host tissues, the extent of the granulomatous response, and the degree of infectivity of the parasite strain are key factors in the pathogenesis of schistosomiasis [3].

The diverse behavior of different strains of *Schistosoma mansoni* [4–11] may account for the regional variation observed in clinical schistosomiasis [12]. The degree of morbidity in human schistosomiasis varies regionally, possibly due to variations in parasite infectivity and fecundity [13].

Previous studies have demonstrated a difference in the development of the BH and SJ strains of *S. mansoni* in the definitive host [4, 6, 7, 14–16]. More hepatic granulomas were observed in mice infected with the *S. mansoni *BH strain, and a significantly lower percentage of mice survived in this group than in the group of mice that were infected with the SJ strain. In mice, fewer parasites of the BH strain were necessary to obtain equivalent pathogenesis than those with the SJ strain [6]. These experiments demonstrated an increased pathogenicity of the BH strain [6, 10, 17, 18]. In areas that are considered endemic for the BH strain, there are frequent cases of decompensated hepatosplenomegaly, which does not occur in regions where the SJ strain is present. Instead, almost all cases of infection with the SJ strain are asymptomatic, with the exception of rare cases of compensated hepatosplenomegaly [19, 20]. In addition, adult worms and eggs of the BH strain are typically larger than those of the SJ strain [10, 14, 21–27].

 A number of factors seem to be fundamentally important for the development of severe forms of schistosomiasis: the number of eggs and the antigens they released; the reinfections; the genetic influence of the host, the hosts immune response to the formation of granulomas, the development of periportal fibrosis and factor modulators, and the associations with aggravating factors such as alcoholism, malnutrition, and hepatitis (particularly hepatitis B and C) which compromise the liver [28]. Mice were experimentally infected and submitted to a low-protein diet using the BH strain of *S. mansoni*. In spite of the reduced number of hepatic granulomas, as well as a reduction in the size of the granuloma, mortality rates among the animals were high [29]. Recent studies [30] used different strains of mice and established an association between malnutrition and the development of hepatic fibrosis. In the state of Minas Gerais (Brazil), the hepatosplenic form of schistosomiasis in children was strongly associated with bathing in streams [31]. In parts of the state of São Paulo (Brazil), characterized by low endemicity, where the main risk factor for *S. mansoni *infection is from leisure in water, there is a significant correlation between the intensity of the infection and the prevalence. The infection rate of the intermediate host *B. tenagophila* was 0.4% in this case [32]. All of these associated factors indicated that the evolution of mansoni schistosomiasis should be seen as a multidisciplinary phenomenon and individual analysis of each case should be performed to gain a better understanding of the infection. 

Considering the importance of *S. mansoni* eggs in the pathogenicity of schistosomiasis and the fact that the induced granulomatous immune response is stage specific [33], the aim of the present study was to investigate the inflammatory response in the lung tissue of mice inoculated in the caudal vein with eggs of the *S. mansoni *BH and SJ strains to determine whether differences existed between the two strains in terms of the inflammatory response around eggs.

## 2. Materials and Methods

 The present study received approval from the Animal Experimentation Ethical Committee of the UNICAMP (Comissão de Ética na Experimentação Animal-CEEA-IB-UNICAMP) under protocol number 870-1.

### 2.1. *S. mansoni* Strains and Egg Collection

 Two *S. mansoni* strains were used in the present study: the SJ strain from São José dos Campos (SP, Brazil), which was maintained in populations of sympatric *B. tenagophila *and the BH strain, originally from Belo Horizonte (MG, Brazil), which was maintained in populations of sympatric *B. glabrata*. Cercariae obtained from the snails were used to infect Swiss SPF (specific pathogen free) mice [34]. The mice were exposed to 100 cercariae for two hours. After this time period had elapsed, the cercariae that remained in the test tubes, where the tails of the mice were immersed, were counted. 

 The eggs from both strains (BH and SJ) of *S. mansoni *were obtained from the intestinal wall of infected mice. Mice were inoculated in the caudal vein with approximately 1000 mature eggs in 0.3 mL of saline solution [35].

### 2.2. Experimental Groups

 Four experimental groups were established in the present study. Group I contained 12 mice inoculated with eggs from the *S. mansoni* BH strain. Group II contained 12 mice inoculated with eggs from the *S. mansoni* SJ strain. Group III contained 12 mice infected percutaneously with 100 cercariae of the *S. mansoni* BH strain 8 weeks prior to being inoculated in the caudal vein with eggs from the *S. mansoni* BH strain. Group IV contained 12 mice infected percutaneously with 100 cercariae of the *S. mansoni* BH strain 8 weeks prior to being inoculated in the caudal vein with eggs from the *S. mansoni* SJ strain.

Two to three animals were used to obtain the mature eggs of the BH strain to be inoculated in the caudal vein of mice. Five to six previously infected animals were used to obtain mature eggs of the SJ strain. The mice in each group were euthanized by cervical dislocation 1, 8, 15, or 34 days after inoculation with the eggs. On each of these preestablished days, 3 mice from each group were euthanized. The worms were then recovered from the mice in groups III and IV via perfusion of the hepatic portal system [36]. Finally, the recovered worms were separated by gender.

### 2.3. The Number and Size of the Granulomas in the Liver and the Lungs

 In order to count and measure the schistosome granulomas in the liver (Groups III and IV) and in the lungs (Groups I, II, III, and IV) of the euthanized mice, histological slices (5 *μ*m thick) were fixed in Bouin's solution, stained with Masson's trichrome and examined using an optical microscope. The number of granulomas per tissue area (0.984704 mm^2^) and the extent of the granulomatous response were determined using the techniques described by Magalhães et al. [17]. Only granulomas that contained an *S. mansoni *egg at the center were measured. Measurements were performed using Image-Pro Lite software, (version 4.0) for Windows 95/NT/98.

### 2.4. Statistical Analysis

The data were analyzed using SAS software for Windows 8.01, 2000 [37]. 

## 3. Results

Table 1 displays the number of cercariae that effectively penetrated and recovered trematodes from Groups III and IV. There was no significant difference between the number of penetrating cercariae in the two groups (*P* = 0.4667). The number of trematodes was significantly higher in Group III than in Group IV (*P* = 0.0012, *P* = 0.0036, *P* = 0.0019; male, female, and total number of trematodes, resp.).

### 3.1. Granuloma Area in the Lung (Table 2, Figures 1, 2, 3, and 4)

**Figure 1:**
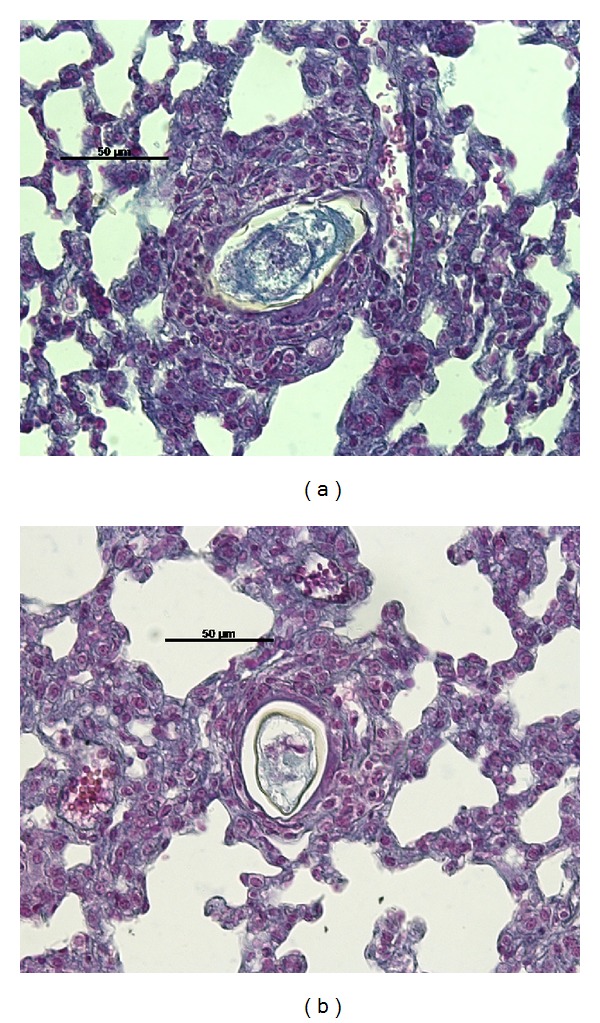
Formation of a halo cell around mature (a) *S. mansoni* BH (Group I) and (b) SJ (Group II) eggs in the pulmonary parenchyma one day after inoculation of the eggs in the caudal vein of mice.

Table 2 displays the number of pulmonary granulomas found in each of the experimental groups.  Figure 1 displays the beginning of the granulomatous reaction one day after inoculation of the BH eggs (Group I) and the SJ eggs (Group II).

**Figure 2:**
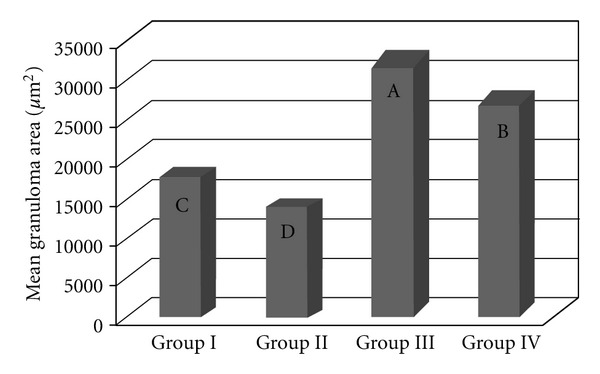
The granuloma area in the lungs of mice that were inoculated with eggs from the *S. mansoni* BH (Group I) or SJ (Group II) strains and of those that were previously infected with the *S. mansoni* BH strain and then inoculated 8 weeks later with eggs from the *S. mansoni* BH (Group III) or SJ (Group IV) strains. The means with different letters exhibited a significant difference (*α* = 0.05).

 The area covered by granulomas differed significantly between the groups, independently of the time since inoculation (*P* < 0.0001). The largest granulomas occurred in Groups III and IV (previously infected with the *S. mansoni* BH strain) and the smallest granulomas were found in Groups I and II (inoculated with eggs from the BH and SJ strains, resp.) (Figure 2). The granulomatous area around the eggs from the BH (Group I) strain was significantly larger than that of the SJ (Group II) strain (Figure 2). The area of the granulomas altered throughout the postinoculation period (*P* < 0.0001), with the smallest granulomas recorded 1 day after inoculation and the largest granulomas recorded after 15 days (Figure 2). Fifteen days after inoculation, the BH eggs had induced a significantly larger granulomatous response than the SJ eggs (Figure 4). The mice previously infected with the *S. mansoni* BH strain (Groups III and IV) exhibited larger granulomas in the lung than the mice in Groups I and II (Figure 2). There was also a significant difference in granuloma size 8 days (*P* < 0.0001) and 34 days (*P* < 0.0001) after inoculation with the eggs (Figure 4). The comparison of mice infected with the *S. mansoni* BH strain and subsequently inoculated with the BH (Group III) or SJ (Group IV) eggs revealed that the granuloma area surrounding BH eggs was significantly larger 8 and 15 days after inoculation (Figure 4).

### 3.2. The Number of Lung Granulomas (Figures 5 and 6)

There were significantly (*P* < 0.0001) more granulomas in the lungs of the mice infected and inoculated with eggs from the BH strain (Group III) (Figure 5). There were no significant differences in the number of granulomas among the other groups (I, II, and IV). Most granulomas were observed 8 and 34 days after inoculation, and the fewest granulomas were found on the first day after inoculation with the eggs. Fifteen days after inoculation, there were no significant differences in the number of granulomas between the four experimental groups. Significantly more granulomas formed on the first day after inoculation in the lungs of previously infected animals (Groups III and IV) than in that of naive mice (Groups I and II). Eight days after inoculation, there were significantly fewer granulomas in the mice from Group IV than in the other groups, whereas no significant difference existed between Groups II and III. Thirty-four days after inoculation, there were significantly more granulomas in Group III than in the other groups (Figure 6). 

### 3.3. The Granulomatous Area in the Liver (Figures 7 to 9)

The granulomatous area was assessed in the livers of mice that were previously infected with the *S. mansoni* BH strain (Groups III and IV). No significant difference was found between the two groups in terms of the area covered by hepatic granulomas (*P* = 0.7412) (Figure 7), even when the time after infection was considered (Figure 8). In both groups, the hepatic granulomatous area increased 8 and 15 days after infection (*P* = 0.0010) and then decreased 34 days after inoculation (Figure 9).

### 3.4. The Number of Granulomas in the Liver (Figures 10 and 11)

Significantly more granulomas were observed in the livers of mice from Group III than in the livers of mice from Group IV (*P* < 0.0001) (Figure 10). In addition, more granulomas were found 1 and 8 days after inoculation (*P* < 0.0001). At these time points, there were significantly more granulomas in mice from Group III than in mice from Group IV (Figure 11).

## 4. Discussion

**Figure 3:**
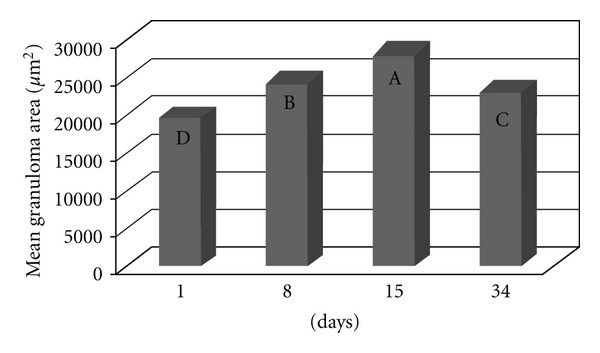
The area of the granulomatous response around the eggs of *S. mansoni* in the lungs of the mice in all 4 experimental groups (I, II, III, and IV) is depicted based on the time since the inoculation of the eggs in the caudal vein. The means with different letters exhibited a significant difference (*α* = 0.05).

**Figure 4:**
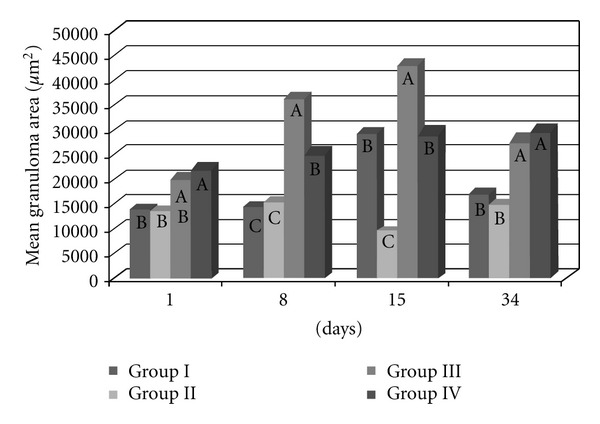
The mean granuloma area in the lungs of the mice that were inoculated with eggs of the *S. mansoni* BH (Group I) or SJ (Group II) strains and of those that were previously infected with the *S. mansoni* BH strain and then inoculated 8 weeks later with eggs from the *S. mansoni* BH (Group III) or SJ (Group IV) strains. The mice were euthanized 1, 8, 15, and 34 days after inoculation with the eggs. At each time point, the means depicted with the same letter do not differ significantly (*α* = 0.05).

**Figure 5:**
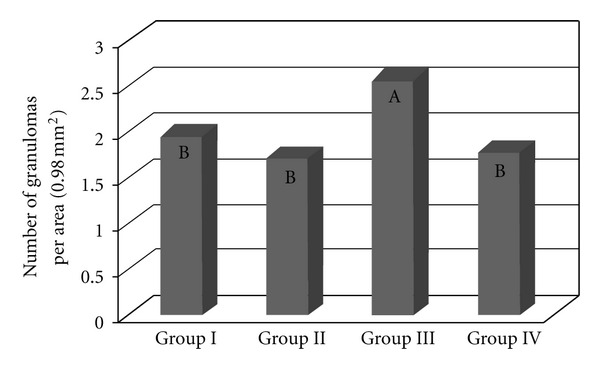
The number of lung granulomas of the mice that were inoculated with eggs of the *S. mansoni* BH (Group I) or SJ (Group II) strains and of those that were previously infected with the *S. mansoni* BH strain and inoculated 8 weeks later with eggs from the *S. mansoni* BH (Group III) or SJ (Group IV) strains. The means with different letters exhibited a significant difference (*α* = 0.05).

**Figure 6:**
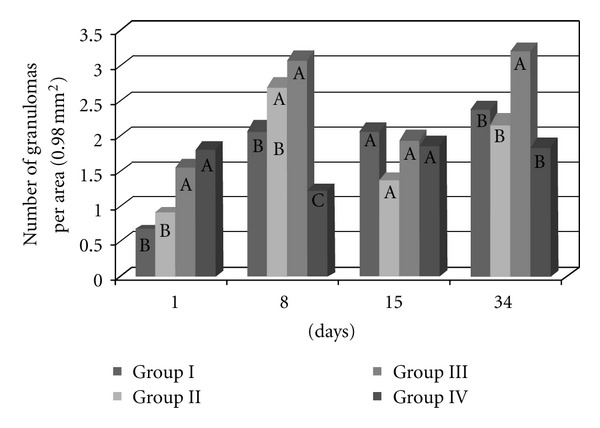
The number of lung granulomas of the mice that were inoculated with eggs of the *S. mansoni* BH (Group I) or SJ (Group II) strains and of those that were previously infected with the *S. mansoni* BH strain and then inoculated 8 weeks later with eggs from the *S. mansoni* BH (Group III) or SJ (Group IV) strains. The mice were euthanized 1, 8, 15 or 34 days after inoculation with the eggs. At each time point, the means depicted with the same letter do not differ significantly (*α* = 0.05).

**Figure 7:**
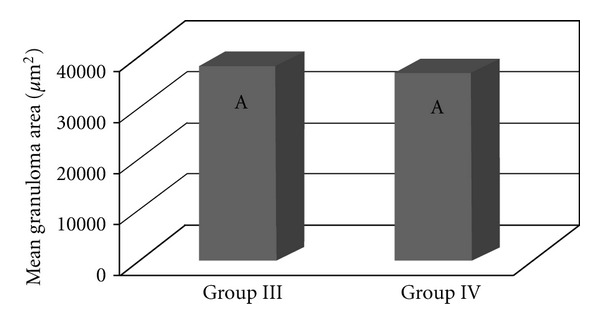
The total granulomatous area in the liver of the mice that were previously infected with the *S. mansoni* BH strain and then inoculated 8 weeks later with eggs from the *S. mansoni* BH (Group III) or SJ (Group IV) strains. The means with same letter do not differ significantly (*α* = 0.05).

**Figure 8:**
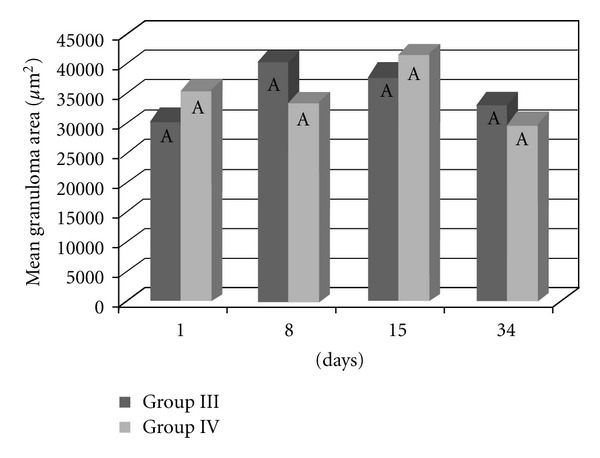
The granulomatous area in the liver of the mice that were previously infected with the *S. mansoni* BH strain and then inoculated 8 weeks later with eggs from the *S. mansoni* BH (Group III) or SJ (Group IV) strains. The mice were euthanized 1, 8, 15, or 34 days after inoculation with the eggs. At each time point, the means depicted with the same letter do not differ significantly (*α* = 0.05).

 The granulomatous response induced by the eggs of *S. mansoni* is a protective mechanism initiated by the host organism, although its appearance is also responsible for the disease pathology. The degree of the response by the host organism depends on the stimulating capacity of the parasite and the integrity of the host immune system. According to Lichtenberg [38, 39], the length and size of the granuloma are proportional to the persistence of the egg in the lesion and the ability of the host cells to destroy antigens. The granulomatous reaction plays an important role in protecting host tissues to sequester antigens released by the eggs, while at the same time causing the pathogenesis [39]. The granulomatous response that was induced in the lungs by the BH eggs (Group I and III) was greater than that induced by the SJ eggs (Groups II and IV) (Figure 2). The increased granulomatous response in the lungs was observed 15 days after inoculation with the eggs (Figure 3). Among all of the groups of the present study, the largest granulomas were observed around the BH eggs in mice that were previously infected with the *S. mansoni *BH strain 8 and 15 days after inoculation in the caudal vein (Figure 4). The response around the SJ eggs, after 15 days in mice without prior infection of the *S. mansoni *BH (Group II) and after 8 and 15 days in mice that were previously infected with the *S. mansoni *BH strain (Group IV), was lower than the responses around eggs of the BH strain. As the number of eggs inoculated in the caudal vein was similar for all groups and there was a similar number of granulomas around both BH and SJ eggs (Groups I and II, Figure 5), the larger size of the BH eggs, which has been reported by several authors [26, 27], appears to influence the extent of the granulomatous response in the lung. Infecting mice with cercariae from the BH strain before they were inoculated with eggs (BH or SJ strain, Groups III and IV) induced a more robust granulomatous response around the eggs in the lungs of infected animals. However, the reaction was significantly lower around SJ eggs, which indicated that the antigenic identity of the eggs from each strain had unique aspects (Figure 2). The granulomatous response in the lungs around the BH eggs (Group III) was significantly greater than the reaction around the SJ eggs (Group IV) 8 and 15 days after inoculation (Figure 4), suggesting a specificity of the host response against the two strains. Although demonstrating host immunological integrity, the large granulomatous responses around the *S. mansoni* eggs compromized the host tissues and blood circulation. The granuloma-inducing antigens, which were secreted and/or excreted by the eggs, were located below the mature eggshell that surrounded the miracidium [40, 41]. Therefore, larger eggs, such as those of the BH strain, could produce an increased amount of antigen. Schramm et al. [41] reported that the stimulation of the immune response by egg antigens was dose dependent. The greater size of the BH eggs (in relation to the SJ eggs) would constitute a greater antigenic volume to be processed by the defense mechanisms of the host and could result in a greater retention of antigenic stimulus, as well as the actual mechanical effect represented by its greater volume. Morphometric and biological differences were also found in the Japanese and Formosan strains of *S. japonicum*. The resistance to infection was greater when the animals were initially exposed to the Formosan strain as opposed to the Japanese strain [42]. A study of the protein composition of mature and non-mature *S. mansoni* eggs of the Porto Rico strain identified a different composition depending on the stage of development, the miracidium, and the hatching fluid [43]. Differences in antigenicity were found between eggs that were inoculated and eggs that were produced by worms in active infections [44]. Even though mature eggs were used in the present study, it is possible that antigens could have been lost during attainment and handling of the eggs and tissues and during the inoculation of the caudal vein. The vast behavioral diversity of *S. mansoni*, with its various developmental stages in both vertebrate and intermediate hosts, is a reflection of the large number of genes and their different expression patterns during each stage of the life cycle [45]. One could hypothesize that the *S. mansoni* SJ strain is different because *B. tenagophila* is the natural intermediate host. Geographical strains of *S. mansoni *have significant pathogenic differences stemming from the degree of organ impairment, which is dictated by the distribution of *S. mansoni* eggs [9], the number of eggs produced by the parasite [6, 46], and the degree of susceptibility of the snail vector [9, 10, 18].

**Figure 9:**
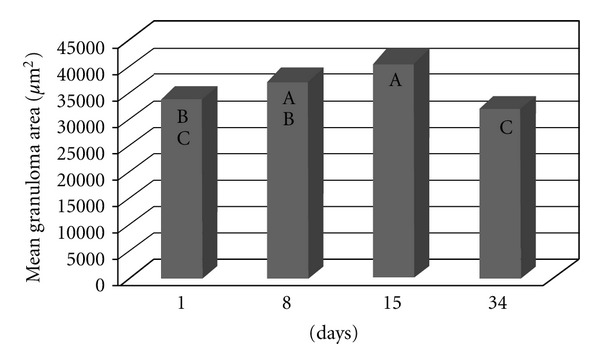
The granulomatous area in the liver of the mice that were previously infected with the *S. mansoni* BH strain and then inoculated 8 weeks later with eggs from the *S. mansoni* BH (Group III) or SJ (Group IV) strains. The mice were euthanized 1, 8, 15, or 34 days following inoculation with the eggs. The means depicted with the same letter do not differ significantly (*α* = 0.05).

**Figure 10:**
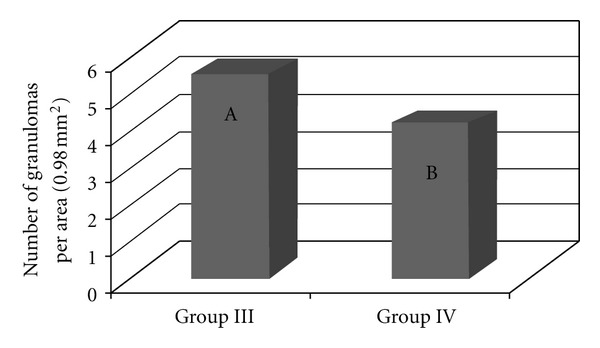
The number of granulomas in the liver of the mice that were previously infected with the *S. mansoni* BH strain and then inoculated 8 weeks later with eggs from the *S. mansoni* BH (Group III) or SJ (Group IV) strains. The means depicted with different letters differ significantly (*α* = 0.05).

**Figure 11:**
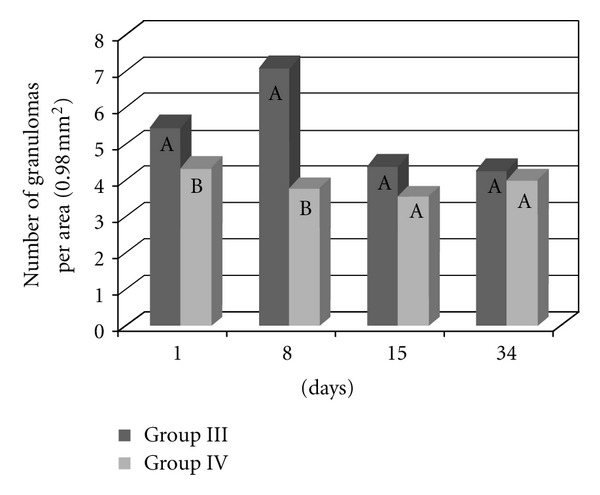
The number of granulomas in the liver of the mice that were previously infected with the *S. mansoni* BH strain and then inoculated 8 weeks later with eggs from the *S. mansoni* BH (Group III) or SJ (Group IV) strains. The mice were euthanized 1, 8, 15, or 34 days after the inoculation with the eggs. For each time point, the means depicted with the same letter do not differ significantly (*α* = 0.05).

 The increased number of granulomas in the lungs and the liver observed in the animals from Group III (infection with *S*.* mansoni* and inoculation with BH eggs) can be attributed to the greater number of eggs resulting from the greater number of worms recovered (Table 1). Granulomatous pulmonary reactions around the eggs were found in mice infected with the BH strain [47]. The granulomas observed in the livers of the mice in Groups III and IV were probably caused by eggs laid by the BH trematodes, since an examination of the livers of the mice in Groups I and II (inoculated with the BH and SJ eggs only) did not reveal the presence of granulomas. The smaller hepatic granulomas found 90 days after infection (34 days after inoculation, Figure 9) resulted from the immunomodulation of the inflammatory response of the eggs during chronic schistosomiasis [48]. The results of the present study showed that granulomas covered a larger area in the lungs of mice that were previously infected with cercariae and subsequently inoculated with eggs from the BH strain. These results indicated that specific granulomatous responses occurred following an infection with the BH and SJ strains of *S. mansoni*.

## Figures and Tables

**Table 1:** The table presents the mean number of cercariae and male and female trematodes that were recovered from mice exposed to 100 cercariae of the *S. mansoni* BH strain and inoculated 8 weeks later with eggs from the *S. mansoni* BH (Group III) or SJ (Group IV) strains. The mice were euthanized 1, 8, 15, and 34 days after being inoculated with eggs in the caudal vein.

Group	Infecting cercariae	Female	Male	Total trematodes	Duncan's test
III	94.90				A
IV	95.00				A

III		17.75			A
IV		9.10			B

III			18.87		A
IV			8.50		B

III				36.62	A
IV				17.60	B

There is no significant difference between means with the same letter (*α* = 0.05).

**Table 2:** Number of pulmonary granulomas recorded, with the mean and standard deviation values from the different groups of mice, which were euthanized after 1, 8, 15, and 34 days.

Groups	Days	Number of observations	Mean area (*μ*m^2^)	Standard deviation
I	1	8	13908.4392	10297.1036
	8	35	14455.5974	8873.95994
	15	12	30432.241	11432.0316
	34	36	17290.6786	8834.70254

II	1	8	13558.7594	10318.3571
	8	17	15336.7856	5982.09941
	15	17	9571.12058	4399.63715
	34	27	14944.4057	5278.07681

III	1	13	20343.3065	6016.07255
	8	35	38047.4264	16323.5628
	15	14	45384.2903	21215.4849
	34	53	28413.4525	9918.21986

IV	1	31	22506.9705	9944.37182
	8	15	25709.9754	15222.4076
	15	37	29895.0112	10174.3078
	34	22	30799.1796	15791.2374

## References

[1] Capron A (1998). Schistosomiasis: forty years’ war on the worm. *Parasitology Today*.

[2] World Health Organization—(WHO) Programmes and projects >Neglected Tropical Diseases. http://www.who.int/schistosomiasis/en/index.html,.

[3] Rollinson D, Southgate VR, Rollinson D, Southgate VR (1987). The genus *Schistosoma*: a taxonomic appraisal. *The Biology of Schistosomes from Genes to Latrines*.

[4] Magalhães LA, Carvalho JF (1969). Verificação do número de machos e de fêmeas de *Schistosoma mansoni* capturados em camundongos infectados com duas cepas do helminto. *Revista da Sociedade Brasileira de Medicina Tropical*.

[5] Magalhães LA, Carvalho JF (1973). Desenvolvimento do *Schistosoma mansoni* das linhagens de Belo Horizonte (MG) e de São José dos Campos (SP) em *Mus musculus*. *Revista de Saúde Pública*.

[6] Magalhães LA, Carvalho JF (1976). Sobre o comportamento de duas linhagens de *Schistosoma mansoni* Sambon, 1907. Proposição para método de estudo quantitativo. *Revista da Sociedade Brasileira de Medicina Tropical*.

[7] Paraense WL, Corrêa LR (1963). Variation in susceptibility of Australorbis glabratus to a strain of *Schistosoma mansoni*. *Revista do Instituto de Medicina Tropical de Sao Paulo*.

[8] Paraense WL, Corrêa LR (1978). Differential susceptibility of *Biomphalaria tenagophila* populations to infection with a strain of *Schistosoma mansoni*. *Journal of Parasitology*.

[9] Saoud MFA (1966). The infectivity and pathogenicity of geographical strains of *Schistosoma mansoni*. *Transactions of the Royal Society of Tropical Medicine and Hygiene*.

[10] Zanotti-Magalhães EM, Magalhães LA, Carvalho JF (1993). Relação entre a patogenicidade do *Schistosoma mansoni* em camundongos e susceptibilidade do molusco vetor. II. Número de ovos nas fezes e número e tamanho dos granulomas nas vísceras. *Revista de Saúde Pública*.

[11] Zanotti-Magalhães EM, Magalhães LA, Carvalho JF (1997). Relação entre a patogenicidade do *Schistosoma mansoni* em camundongos e susceptibilidade do molusco vetor. IV. Infecciosidade dos miracídios. *Revista de Saúde Pública*.

[12] Coelho PMZ, Raso P, Mello RT, Toppa NH (1989). Dimensões do granuloma hepático produzido por ovos de duas linhagens geográficas do *Schistosoma mansoni*, no camundongo. *Memórias do Instituto Oswaldo Cruz*.

[13] Higgins-Opitz SB, Dettman CD (1991). The infection characteristics of a South African isolate of *Schistosoma mansoni*: a comparison with a Puerto Rican isolate in BALB/c mice and *Mastomys coucha*. *Parasitology Research*.

[14] Magalhães LA, Carvalho JF (1973). Estudo morfológico de *Schistosoma mansoni* pertencentes a linhagens de Belo Horizonte (MG) e de São José dos Campos (SP). *Revista de Saúde Pública*.

[15] Magalhães LA, Alcantara FG, Carvalho JF (1975). Alguns dados referentes ao estudo parasitológico e anatomopatológico de duas linhagens de *Schistosoma mansoni*, Sambon, 1907. *Revista de Saúde Pública*.

[16] Yoshioka L, Zanotti-Magalhaes EM, Magalhães LA, Linhares AX (2002). *Schistosoma mansoni*: a study of pathogenesis of Santa Rosa strain (Campinas, SP, Brasil) in mice. *Revista da Sociedade Brasileira de Medicina Tropical*.

[17] Magalhães LA, Alcantara FG, Carvalho JF (1975). Distribuição de lesões esquistossomóticas extra-hepáticas em camundongos infectados pelas linhagens BH e SJ de *Schistosoma mansoni*. *Revista de Saúde Pública*.

[18] Zanotti-Magalhães EM, Magalhães LA, Carvalho JF (1995). Relação entre a patogenicidade do *Schistosoma mansoni* em camundongos e susceptibilidade do molusco vetor. III. Mortalidade, peso corporal e das vísceras. *Revista de Saúde Pública*.

[19] Dias LCS, Glasser CM, Etzel A (1988). The epidemiology and control of schistosomiasis mansoni where *Biomphalaria tenagophila* is the snail host. *Revista de Saúde Pública São Paulo*.

[20] Santos NR (1967). *Esquistossomose mansônica autóctone no Vale do Médio Paraíba, Estado de São Paulo, Brasil: contribuicão para o estudo da zona endêmica [Ph.D. thesis]*.

[21] Kastner MRQ, Kohn A, Teixeira ED, Pitanga LC (1975). Estudo morfológico do *Schistosoma mansoni* Sambon, 1907, encontrado na espécie humana. *Revista da Sociedade Brasileira de Medicina Tropical*.

[22] Machado-Silva JR, Galvão C, Presgrave OAF, Rey L, Gomes DC (1994). Host-induced morphological changes of *Schistosoma mansoni* Sambon, 1907 male worms. *Memorias do Instituto Oswaldo Cruz*.

[23] Machado-Silva JR, Galvão C, de Oliveira RM, Presgrave OA, Gomes DC (1995). *Schistosoma mansoni* Sambon, 1907: comparative morphological studies of some Brazilian strains. *Revista do Instituto de Medicina Tropical de São Paulo*.

[24] Machado-Silva JR, Lanfred RM, Gomes DC (1997). Morphological study of adult male worms of *Schistosoma mansoni* Sambon, 1907 by scanning electron microscopy. *Memórias do Instituto Oswaldo Cruz*.

[25] Machado-Silva JR, Pelajo-Machado M, Lenzi HL, Gomes DC (1998). Morphological Study of Adult Male Worms of *Schistosoma mansoni* Sambon, 1907 by Confocal Laser Scanning Microscopy. *Memorias do Instituto Oswaldo Cruz*.

[26] Neves RH, Dos Santos Pereira MJ, De Oliveira RMF, Gomes DC, Machado-Silva JR (1998). *Schistosoma mansoni* Sambon, 1907: morphometric differences between adult worms from sympatric rodent and human isolates. *Memorias do Instituto Oswaldo Cruz*.

[27] Paraense WL, Corrêa LR (1981). Observations on two biological races of *Schistosoma mansoni*. *Memorias do Instituto Oswaldo Cruz*.

[28] Prata A, Coura JR, Carvalho OS, Coelho PMZ, Lenzi HL (2008). Fases e formas clínicas da esquistossomose mansoni. *Schistosoma Mansoni & Esquistossomose. Uma Visão Multidisciplinar*.

[29] Magalhães LA, Guaraldo AMA, Zanotti-Magalhães EM, Carvalho JF, Sgarbieri VC, Alcântara FG (1986). Esquistossomose mansônica em camundongos experimentalmente subnutridos. *Revista de Saúde Pública*.

[30] Coutinho EM (2004). Malnutrition and hepatic fibrosis in murine schistosomiasis. *Memorias do Instituto Oswaldo Cruz*.

[31] Lima E, Costa MF, Rocha RS, Magalhães MHA, Katz N (1994). Um modelo hierárquico de análise das variáveis sócio-econômicas e dos padrões de contatos comáguas associados à forma hepatoesplênica da esquistossomose. *Cadernos de Saúde Pública*.

[32] Dias LCS, Glasser CM, Marçal O, Bonesso PI (1994). Epidemiologia da esquistossomose mansônica em área de baixa endemicidade. *Cadernos de Saúde Pública Rio de Janeiro*.

[33] Warren KS, Domingo EO, Cowan RB (1967). Granuloma formation around *Schistosome* eggs as a manifestation of delayed hypersensitivity. *American Journal of Pathology*.

[34] Magalhães LA (1969). Technic of viability evaluation of *Schistosoma mansoni*’s cercariae penetration in *Mus musculus*. *Hospital*.

[35] Warren KS, Domingo EO (1970). *Schistosoma mansoni*: stage specificity of granuloma formation around eggs after exposure to irradiated cercariae, unisexual infections, or dead worms. *Experimental Parasitology*.

[36] Yolles TK, Moore PV, Degensti DL, Ripson CA, Meleney HE (1947). A technique for the perfusion of laboratory animals for the recovery of schistosomes. *Journal of Parasitology*.

[37] Institute SAS (2000). *SAS System for Windows, Version 8. 01*.

[38] Lichtenberg FV (1962). Host response to eggs of *S. mansoni*. I. Granuloma formation in the unsensitized laboratory mouse. *American Journal of Pathology*.

[39] Lichtenberg FV (1968). Studies on granuloma formation. III. Antigen sequestration and destruction in the schistosome pseudotubercle. *American Journal of Pathology*.

[40] Ashton PD, Harrop R, Shah B, Wilson RA (2001). The schistosome egg: development and secretions. *Parasitology*.

[41] Schramm G, Gronow A, Knobloch J (2006). IPSE/alpha-1: a major immunogenic component secreted from *Schistosoma mansoni* eggs. *Molecular and Biochemical Parasitology*.

[42] Sadun EH, Yamaki A, Lin SS, Burke JC (1961). Studies on the host-parasite relationships to *Schistosoma japonicum*. VI. Acquired resistance in mice and monkeys infected with the Formosan and Japanese strains. *The Journal of Parasitology*.

[43] Mathieson W, Wilson RA (2010). A comparative proteomic study of the undeveloped and developed *Schistosoma mansoni* egg and its contents: the miracidium, hatch fluid and secretions. *International Journal for Parasitology*.

[44] Eltoum IA, Wynn TA, Poindexter RW (1995). Suppressive effect of interleukin-4 neutralization differs for granulomas around *Schistosoma mansoni* eggs injected into mice compared with those around eggs laid in infected mice. *Infection and Immunity*.

[45] Anderson L, Pierce RJ, Verjovski-Almeida S (2012). *Schistosoma mansoni* histones: from transcription to chromatin regulation: an in *silico* analysis. *Molecular & Biochemical Parasitology*.

[46] Bina JC, Prata A (2003). Esquistossomose mansônica na área hiperendêmica de Taquarendi. I—Infecção pelo *Schistosoma mansoni* e formas graves. *Revista da Sociedade Brasileira de Medicina Tropical*.

[47] Zanotti EM, Magalhães LA, Piedrabuena AE (1983). Evaluation of the pathogenicity resulting from infection by *Schistosoma mansoni* Sambon 1907, agent of unisexual infections in *Mus musculus*. *Revista de Saude Publica*.

[48] Andrade ZA, Warren KS (1964). Mild prolonged schistosomiasis in mice. Alterations in host response with time and the development of portal fibrosis. *Transactions of the Royal Society of Tropical Medicine and Hygiene*.

